# Experimental Model of Saccular Abdominal Aortic Aneurysm in Swines with Pericardium Sac

**DOI:** 10.5935/1678-9741.20160005

**Published:** 2016

**Authors:** Maurício de Amorim Aquino, Svetlana Maria Wanderley de Barros, Aldemar Araújo Castro, Guilherme Benjamin Brandão Pitta, Adamastor Humberto Pereira

**Affiliations:** 1 Universidade Federal do Rio Grande do Sul (UFRGS), Porto Alegre, RS, Brazil.; 2 Vascular Surgery Service and Angiology and Vascular Surgery Service of Hospital Santa Izabel/Santa Casa de Misericórdia Bahia, Salvador, BA, Brazil.; 3 Universidade Estadual de Ciências da Saúde de Alagoas (UNCISAL), Maceió, AL, Brazil.; 4 Center of Experimental Surgery and Bioterium of Universidade Estadual de Ciências da Saúde de Alagoas (UNCISAL), Maceió, AL, Brazil.; 5 Universidade Federal do Rio Grande do Sul Medical School (FAMED-UFRGS), Porto Alegre, RS, Brazil.

**Keywords:** Aneurysm, Aorta, Abdominal, Models, Animal, Pericardium, Swine

## Abstract

**Objective:**

To consider modifications in an experimental model of saccular aortic aneurysm, aiming at better reproducibility, to be used in the development of vascular prostheses.

**Methods:**

Experimental study in two phases, developed in the Center of Experimental Surgery and Bioterium (CCEB) of the University of Health Sciences of Alagoas (UNCISAL), with 11 hybrid swine, female, mean weight of 20 ± 5 kg, according to modifications in the Perini technique was performed. In the first phase, the aneurysm was confectioned with bovine pericardial patch. In the second phase, fifteen days later, the patency of the aneurysms was confirmed by Doppler ultrasonography. The described variables were aortic and aneurysm sac patency, incidence of rupture, morbidity and mortality. The statistical analysis program used was STATA v.8.

**Results:**

All animals survived to the procedures. Surgical mean time was 73 minutes. Aneurysm rupture, proximal or distal aortic thrombosis, visceral or legs ischemia weren't observed. Parietal thrombus formation was observed in all of the aneurysms, two of which (18%; IC 95% = 3.98 - 48.84) were occluded and nine (82%; IC 95% = 51.15 - 96.01) were patent.

**Conclusion:**

In this series, the modifications carried out in the technique related to the surgical approach, race, anesthesia, and imaging exams reproduced the experimental model, reducing its costs, without hindering the analysis of the variables. The satisfactory patency ratio allows the method to be used in experimental models for the development of vascular prostheses.

**Table:** 

**Abbreviations, acronyms & symbols**	
CCEB	= Center of Experimental Surgery and Bioterium
COBEA	= Colégio Brasileiro de Experimentação Animal
PTFE	= Polytetrafluoroethylene
UNCISAL	= University of Health Sciences of Alagoas

## INTRODUCTION

Experimental models reproducing as much as possible the conditions found in the aorta of human beings and preserving its anatomical and physiopathological characteristics are needed for research on the development of endovascular devices^[1]^. Materials used in the correction of aortic endovascular aneurysms are under constant improvement in search of the ideal device for a minimally invasive treatment^[2]^.

Experimental models in animals have been used in vascular and endovascular surgery for decades. The literature is rich in studies proposing models of aortic aneurysms with the used of a myriad of techniques^[3]^. However, the fast pace of advancements in science demands frequent evaluations of established methods in search of improvements and adaptations according to the changing needs of clinical research.

The objective of our study is to propose modifications to an experimental model of aortic aneurysm in swine with bovine pericardial patch (Perini, 2008)^[4]^, aiming at better reproducibility, to be used in the study and development of endovascular prostheses.

## METHODS

This was an experimental study conducted at the Center of Experimental Surgery and Bioterium (CCEB) of the University of Health Sciences of Alagoas (UNCISAL) in Maceió, AL, Brazil.

The project was approved by the Research Ethics Committee of UNCISAL. The ethical principles for animal experimentation of the Brazilian College of Animal Experimentation (Colégio Brasileiro de Experimentação Animal - COBEA)^[5]^, the principles for conducting research with animals (Geneva, 1985)^[6]^, and the Federal Council of Veterinary Medicine Resolution 714/02^[7]^, Decree 24.645/34^[8]^ and Federal Law 9605/98^[9]^ have all been observed.

The sample consisted of 11 Landrace and Large White crossbred swine, female, supplied by the same farmer, properly vaccinated and dewormed, according to their age. The animals were kept in individual stalls with water *ad libitum* and fed with feed, without the addition of lipid supplements, balanced and adapted to their age.

The experiment consisted of two phases. In the first phase, the saccular aneurysm was created using a bovine pericardial patch. In the second phase, 15 days after the first one, patency was confirmed through Doppler ultrasonography, using LOGIQe (GE^®^). In both phases, anesthesia was administered in compliance with the protocol for general anesthesia in swine of the CCEB/UNCISAL.

### Technical anesthesia

The animals were placed under a 12-hour solid food fast and a 3-hour liquid fast and were weighted before pre-anesthetic induction. Anesthesia was administered according to the CCEB/UNCISAL protocol, using subcutaneous atropine, ketamine, and intramuscular midazolam as pre-anesthetic drugs. Next, venoclysis was performed in the marginal ear vein with Jelco 20 catheter for the infusion of liquids and drugs. Fluid replacement was achieved with 0.9% saline solution at 20 ml/kg/hour. Inhalation anesthesia was maintained with halothane and oxygen using a facial mask.

### Surgical procedure

In the first phase, the abdominal aorta was exposed using the transabdominal approach, with a median xiphopubic incision, followed by circumferential dissection of the aorta between the renal arteries and the distal branch (common iliac arteries and internal iliac artery branch).

**Fig. 1:**
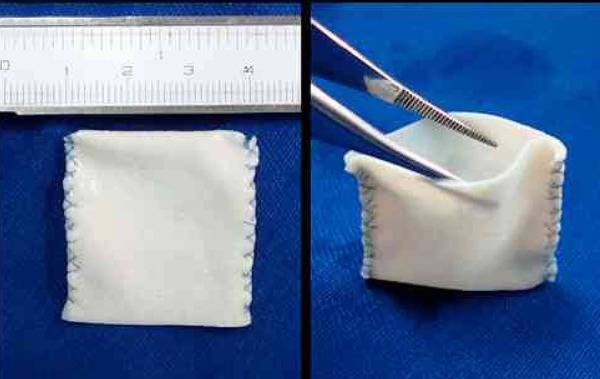
3 x 3 cm bovine pericardial sac for creating the aortic aneurysm.

**Fig. 2:**
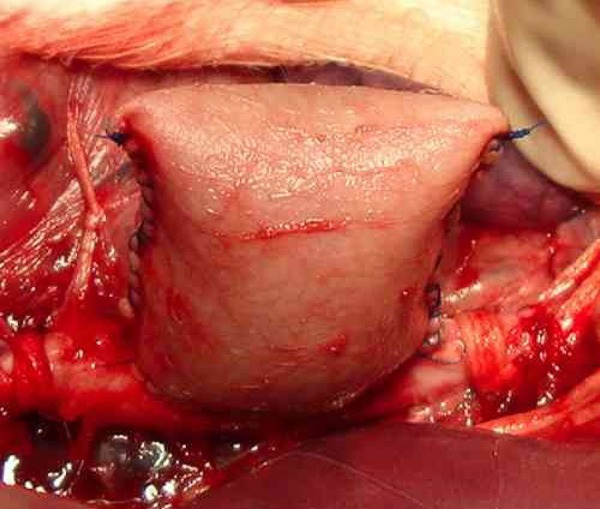
Saccular aneurysm after restoring blood flow.

A 3 cm segment was chosen to make the aneurysm, the branches were repaired with 3.0 linen thread, and intravenous heparin (100UI/kg) was administered. Then, the proximal and distal aorta was clamped to the chosen segment and longitudinal arteriotomy was performed, followed by suturing of the previously made 3x3 cm bovine pericardial patch in the shape of a sac using 6.0 polypropylene continuous suture (Figures 1 and 2). Layered closure of the cavity was performed: retroperitoneum with 3.0 catgut running suture; peritoneum with 2.0 catgut running suture; aponeurosis with 0 mononylon U-suture; skin with 3.0 mononylon Donati suture.

**Fig. 3:**
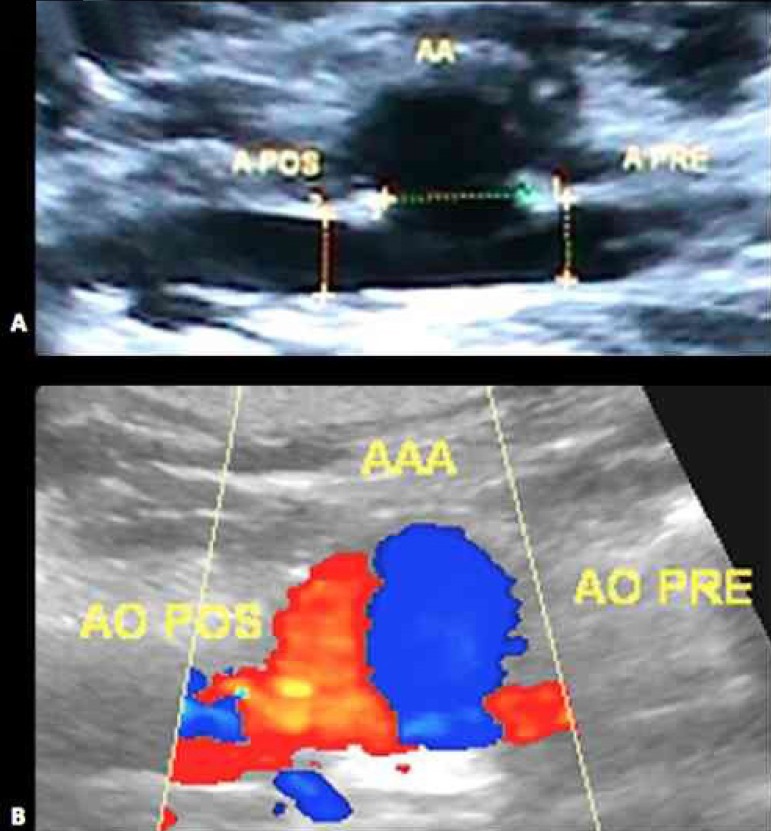
Ultrasonography B mode identifying the aneurysm (AA) and the proximal (A PRE) and distal (A POS) aorta. B - Doppler ultrasonography showing aneurysm patency.

In the second phase, 15 days after the first, Doppler vascular ultrasonography was performed in order to confirm patency of the aneurysms (Figure 3).

### Data Analysis

Descriptive data analysis was performed and it is shown as means and standard deviation and frequency distribution. A 95% confidence interval was observed. The following variables were described: patency of the aorta and aneurysm sac, rupture rate, morbidity and mortality after experimental model was performed. Data was processed using statistics package STATA v.8.

## RESULTS

Eleven Large White and Landrace crossbred swine, female, average weight of 20 ± 5 kg, were operated on. Mean surgical time was 73 minutes, without any complications such as hyperthermia or arrhythmia.

All animals survived to the procedures. No cases of aneurysm rupture, thrombosis of the proximal or distal aorta, ischemia of the paws or intra-abdominal viscera were identified. Average infrarenal aortic clamping time was 17.82 min (IC 95% = 14.96 - 20.68). In the postoperative period, animals maintained normal behavior in their stalls and gained weight until the second phase of the experiment.

Formation of a mural thrombus was observed in all animals, with two of them (18%) showing occlusion of the aneurysm sac. Nine (82%) animals presented patent aneurysm, being eligible to be used in studies for the development of new endovascular prostheses (Table 1).

**Table 1:** Analysis of aneurysm patency.

Aneurysm	N	(%)	IC 95%
Occluded	2	18.18	3.98 - 48.84
Patent	9	81.82	51.15 - 96.01

## DISCUSSION

The advancement of the endovascular technique for the treatment of abdominal aortic aneurysm has brought the need for experimental models to improve related materials and techniques, being the *in vivo* experimentation with animals the most adequate for the execution of these studies^[4]^.

The models adopted should correspond as much as possible to the physiology and physiopathology of humans. Since the similarities in the anatomy and fibrinolytic and coagulation systems of humans and swine are established in the literature - which is less observed for other species such as rabbits, dogs, rats, and mice, the pig was chosen for this study^[10,11]^.

Among the advantages of using swine in cardiovascular research, the following can be highlighted: easy handling; characteristics of the lipid metabolism, lipoprotein profile, and platelet aggregation; formation of thrombus and deposits of fibrin after endothelial lesion; and histological similarity to human neointima. The disadvantages are the rapid weight gain, the cost of maintenance, lower tolerance to anesthesia, and the risk of medullary ischemia with paralysis of the hind paws^[12]^.

Several studies have described models of aortic aneurysm in experimental animals^[1,13-15]^. Among others, of importance are methods such as the use of anterior synthetic patches [polytetrafluoroethylene (PTFE) or Dacron], fascia, peritoneum, intestine, and internal jugular vein; adventitial resection; interposition of the patch; the use of elastase; and transluminal dilation. All of them have advantages and disadvantages, therefore, the researcher must be aware of the characteristics of each technique ^[3,13]^.

In this study, we opted for creating an infrarenal abdominal aortic aneurysm from a bovine pericardial patch according to the model proposed by Perini^[4]^, but with a few modifications.

Bovine pericardium has been used in vascular surgery since 1971, when Ionesco and colleagues used a cardiac valve prosthesis made with bovine pericardium preserved in glutaraldehyde. The purpose of the glutaraldehyde solution is to reduce antigenicity and increase resistance to degradation. Aneurysmal dilation of the pericardial patch may occur, but there are no reports of rupture with other synthetic patches ^[16]^.

In 2008, Perini^[4]^ reported a new abdominal aortic aneurysm model in swine, with a bovine pericardium sac. To that end, 11 Large White female swine, with an average weight of 20 kg, were used. The study was divided into two phases, with a 15-day interval between the creation of the aneurysm and the analysis of its variables (thrombosis, rupture, surgical morbidity and mortality). Our study followed the same procedure, with an equal number of animals, the same gender and weight characteristics, and respecting the 15-day interval between the creation of the aneurysm and the analysis of the variables. However, a few alterations were made in order to simplify the method and further reduce its cost.

In terms of animals, hybrid swine were used, crossbred Large White and Landrace, rather than the usual purebred Landrace or Large White. For the anesthesia, halothane was used for inhalation instead of isoflurane, which lowered the costs. Despite reports in the literature of the incidence of malignant hyperthermia with the use of halothane in Landrace and Large White swine ^[17]^, this was not observed in the hybrid animals used in this study.

The transperitoneal approach was used to access the aorta rather than the retroperitoneal. Since the swine gain weight rapidly, making them difficult to handle when they are bigger, swine with an average weight of 20 kg were used. In these animals, dissection and exposure of the aorta through the retroperitoneal approach is more technically difficult to achieve due to the limited surgical field and small caliber of the vessels. There were no cases of evisceration nor complications related to handling intestinal loops, as described in the literature (cardiac arrhythmia, distension or difficulty to close the abdominal wall).

Patency of the aorta and the aneurysm sac 15 days after it was created was verified with Doppler ultrasonography. This method made it possible to analyze not only the presence of flow inside the aneurysm sac, but also the hemodynamic characteristics of the flow, providing a greater amount of information. In addition, the cost is lower and there is no exposure to radiation, compared to an angiographic study.

Experimental models of aortic aneurysm to be used in the training of endovascular techniques should have as many characteristics as possible in common with those found in humans. Among them, it is essential to have an increase in diameter of 50% compared to the diameter of a normal aorta, patency of lumbar arteries, and stability of the aneurysm allowing for surgical manipulation in a few weeks or months ^[13,18]^. So far, no other model had associated the aforementioned characteristics with other advantages, such as the lower cost, low morbidity and mortality, and easy reproducibility.

The saccular aneurysm model with bovine pericardium proved to be adequate to test endovascular devices, since it mimics a similar situation to that found in aneurysms observed in humans, with proper diameter, patency of the collateral and terminal branches of the lumbar plexus, and partial thrombosis of the wall. Bovine pericardium is commercially available as a patch for arterial repair, in a range of cuts and sizes and cheaper than Dacron and PTFE. The short interval between the creation and maturation of the aneurysm (15 days) allows the model to be used in larger animals without increasing maintenance costs with medication and feed. Surgical time needed to perform this technique was well tolerated by the swine, without the complications described in the literature, such as paralysis of the hind paws, death from anesthetic complications, renal failure, intestinal perforation, sepsis, early rupture, and thrombosis of the aorta or iliac arteries.

## CONCLUSION

Based on this study, we have shown that the technique proposed for the confection of abdominal aortic aneurysm using bovine pericardium has good patency rates and it could be used to prepare experimental models for the development of new endovascular prostheses. The modifications made to the original technique have helped to reproduce the model at a reduced cost, without prejudice to the analysis of the proposed variables.

**Table:** 

**Authors' roles & responsibilities**	
MAA	Conception and study design; execution of operations and/or trials; analysis and/or data interpretation; writing of the manuscript or critical review of its content; final approval of the manuscript
SMWB	Execution of operations and/or trials; final approval of the manuscript
AAC	Conception and study design; analysis and/or data interpretation; final approval of the manuscript
GBBP	Conception and study design; writing of the manuscript or critical review of its content; final approval of the manuscript
AHP	Conception and study design; analysis and/or data interpretation; writing of the manuscript or critical review of its content; final approval of the manuscript
